# Clinical Characteristics and Risk Factors of First-Ever Stroke in Young Adults: A Multicenter, Prospective Cohort Study

**DOI:** 10.3390/jpm12091505

**Published:** 2022-09-14

**Authors:** Yea Jin Jo, Dae Hyun Kim, Min Kyun Sohn, Jongmin Lee, Yong-Il Shin, Gyung-Jae Oh, Yang-Soo Lee, Min Cheol Joo, So Young Lee, Min-Keun Song, Junhee Han, Jeonghoon Ahn, Won Hyuk Chang, Yun-Hee Kim, Deog Young Kim

**Affiliations:** 1 Research Institute of Rehabilitation Medicine, Yonsei University College of Medicine, Seoul 03722, Korea; 2 Department of Rehabilitation Medicine, Yonsei University College of Medicine, Seoul 03722, Korea; 3 Department of Rehabilitation Medicine, College of Medicine, Chungnam National University, Daejon 34134, Korea; 4 Department of Rehabilitation Medicine, Konkuk University School of Medicine, Seoul 05030, Korea; 5 Department of Rehabilitation Medicine, Pusan National University School of Medicine, Pusan National University Yangsan Hospital, Yangsan 46241, Korea; 6 Department of Preventive Medicine, Wonkwang University School of Medicine, Iksan 51538, Korea; 7 Department of Rehabilitation Medicine, Kyungpook National University School of Medicine, Kyungpook National University Hospital, Daegu 41566, Korea; 8 Department of Rehabilitation Medicine, Wonkwang University School of Medicine, Iksan 51538, Korea; 9 Department of Rehabilitation Medicine, Jeju National University Hospital, Jeju National University School of Medicine, Jeju 63243, Korea; 10 Department of Physical and Rehabilitation Medicine, Chonnam National University Medical School, Kwangju 61186, Korea; 11 Department of Statistics, Hallym University, Chunchon 24252, Korea; 12 Department of Health Convergence, Ewha Womans University, Seoul 03760, Korea; 13 Department of Physical and Rehabilitation Medicine, Center for Prevention and Rehabilitation, Heart Vascular Stroke Institute, Samsung Medical Center, Sungkyunkwan University School of Medicine, Seoul 06351, Korea; 14 Department of Health Science and Technology, Department of Medical Device Management and Research, Department of Digital Healthcare, SAIHST, Sungkyunkwan University, Seoul 03063, Korea

**Keywords:** stroke, risk factors, young adults

## Abstract

Stroke in young adults has catastrophic consequences and has increased in prevalence, contrary to the trends of most other diseases. This study aimed to determine the major characteristics and risk factors for stroke in younger adults compared with older adults. From the Korean Stroke Cohort for Functioning and Rehabilitation, 10,584 patients with first-ever stroke between August 2012 and March 2015 were enrolled retrospectively and divided into younger (age ≤ 45) and older groups (age > 45). The clinical characteristics and risk factors of stroke were compared between the younger and older groups. The younger group comprised 915 patients (8.6%). The proportion of hemorrhage strokes in the younger group (42.3%) was significantly higher than in the older group (20.0%) (*p* < 0.001). Obesity, current smoking, and heavy alcohol consumption were significantly more common risk factors in the younger group than in the older group for all stroke types, whereas hypertension, diabetes mellitus, hyperlipidemia, atrial fibrillation, and coronary heart disease were significantly more frequent in the older group (both *p* < 0.001). The major risk factors in the younger group may be lifestyle-related. Therefore, increasing awareness of lifestyle-related risk factors may be necessary to prevent stroke in young adults.

## 1. Introduction

Stroke incidence is age-dependent but has increased in young adults [1] despite an overall decline in stroke hospitalizations and mortality in recent years [2]. The US National Survey of Stroke conducted in 1981 reported that only 3.7% of all strokes occurred in patients aged 15–45 years [3]. A retrospective study in 1990 reported that patients aged 15–45 then accounted for 8.5% of all patients admitted with stroke [4]. Since 2000, studies have reported that approximately 10% of all strokes occur in young adults [5,6]. Recent studies have shown that the incidence of ischemic stroke increased with age; an estimated 10% to 20% of these events occur in young people aged 18 to 50 years [7,8].

This is of great public health significance because strokes in younger patients carry the potential for a greater lifetime burden of disability [9] and may have more catastrophic consequences for people of working age [10]. Stroke at a young age not only results in impairment in basic daily activities but also impacts participation in extended activities, such as returning to work [11]. Stroke causes disability in 60% of survivors, and it is difficult to return to society immediately after the onset of stroke [12]. The increased incidence of stroke in young adults is expected to result in significant personal as well as social costs. Thus, it is important to investigate the characteristics and risk factors for stroke in this population.

National cohort studies of ischemic stroke in young adults have been conducted in Europe and the United States, but studies in the East are still scarce [5,13]. In particular, studies of hemorrhagic stroke in young adults have rarely been conducted because of its low incidence compared with ischemic stroke [14]. This study aimed to determine the major clinical characteristics and risk factors of stroke in young adults compared with older adults through a retrospective study of the Korean Stroke Cohort for Functioning and Rehabilitation (KOSCO), a large, multicenter, prospective cohort of acute first-ever stroke patients admitted to participating hospitals in nine distinct areas of Korea [12].

## 2. Materials and Methods

### 2.1. Study Design and Participants

**Figure 1:**
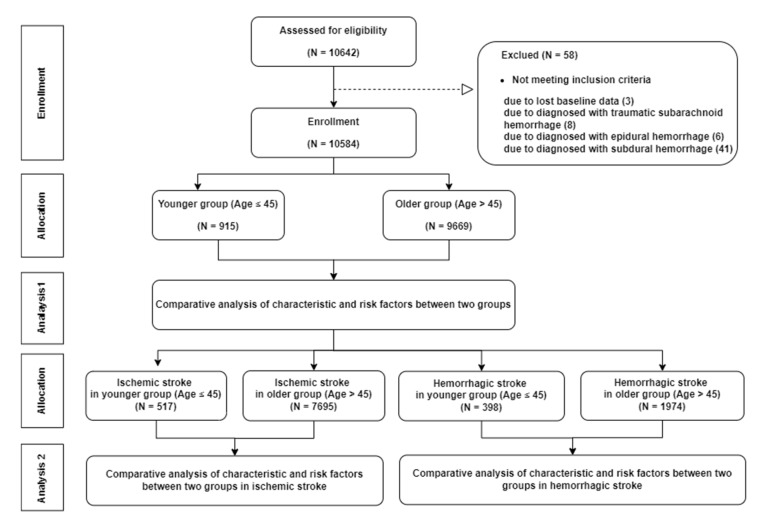
Flow chart of study population.

The study population included 10,584 patients enrolled in the KOSCO study. We screened 10,642 patients who registered with KOSCO from August 2012 to March 2015 and excluded 3 patients with lost data, 8 patients with traumatic subarachnoid hemorrhage, 6 patients with epidural hemorrhage, and 41 patients with subdural hemorrhage. (Figure 1). The inclusion criteria of KOSCO were (1) first-ever acute stroke, (2) age ≥ 19 years at onset of stroke, and (3) onset of symptoms within 7 days prior to inclusion. The exclusion criteria were (1) transient ischemic attack, (2) history of stroke, (3) traumatic intracerebral hemorrhage, and (4) not Korean. All patients were diagnosed with stroke through neuroimaging, such as MRI, CT, CT angiography, or MR angiography, according to standard clinical practice. Neuroimaging was reviewed by neuroimaging specialists in each institute. The rationale and protocols of the KOSCO study were described in an earlier study [15]. We divided the patients into a younger group (age ≤ 45) and an older group (age > 45). The age cut-off for defining young adult stroke varied between 30 and 65 years in previous studies [5,16]. We chose a cut-off age of 45 years, following similar studies that did the same [4,17,18]. We analyzed the baseline characteristics and risk factors for ischemic stroke and hemorrhagic stroke separately and compared them between the younger and older groups. Written informed consent was obtained from all patients prior to inclusion in the study, and the study protocol was approved by the ethics committee of each hospital.

### 2.2. Baseline Characteristics

Certified physicians conducted a complete enumeration survey of all patients at baseline by reviewing medical records at first admission. Survey items included demographic data, such as age and sex, as well as duration from onset to emergency room (ER), affected side, stroke location, vascular abnormality, pre-stroke modified Rankin Scale (mRS) [19], Charlson Comorbidity Index [20], and initial stroke severity. Initial stroke severity was checked at the time of hospital arrival using the National Institutes of Health Stroke Scale (NIHSS) [21] for ischemic stroke or the Glasgow Coma Scale (GCS) [22] for hemorrhagic stroke. Stroke type was divided into ischemic stroke and hemorrhagic stroke and was determined using neuroimaging. Hemorrhagic stroke included intracerebral hemorrhage (ICH), subarachnoid hemorrhage (SAH), and intraventricular hemorrhage (IVH).

### 2.3. Risk Factors

Cerebrovascular risk factors were assessed by certified physicians using standardized, structured questionnaires and were classified according to the current guidelines of the American Heart Association [23]. Risk factors for stroke in this study included hypertension, diabetes mellitus, coronary heart disease, atrial fibrillation, left ventricular hypertrophy, peripheral artery disease, hyperlipidemia, low cholesterol, obesity, smoking, alcohol, and family history. Hypertension was defined as a clinical diagnosis of hypertension, current use of an anti-hypertension drug, systolic blood pressure ≥ 140 mm Hg, or diastolic blood pressure ≥ 90 mm Hg. Diabetes mellitus was defined as a clinical diagnosis of diabetes mellitus, current use of an oral hyperglycemic agent or insulin, or a fasting glucose level > 126 mg/dL. Coronary heart disease was defined as a history of coronary heart disease or a diagnosis of coronary heart disease by EKG, coronary angiography, coronary MRI, or echocardiography at admission. Atrial fibrillation was defined as a history of atrial fibrillation or a diagnosis of atrial fibrillation by EKG or echocardiography at admission. Left ventricular hypertrophy was defined as a history of left ventricular hypertrophy, left ventricular ejection fraction ≤ 50% on echocardiography, or left ventricular ejection fraction ≤ 40% on radionuclide ventriculography. Hyperlipidemia was defined as a history of hyperlipidemia, past use of medication for hyperlipidemia, low-density lipoprotein ≥ 160 mg/dL, or total cholesterol ≥ 240 mg/dL. Low cholesterol was defined as a history of low cholesterol or total cholesterol < 160 mg/dL. Obesity was defined as a clinical diagnosis of obesity or a body mass index ≥ 25. Current smokers were those who had smoked within 1 year of the survey date, and past smokers were those who quit smoking > 1 year before the survey date. Participants were identified as moderate drinkers if they consumed no more than one can of beer a day or heavy drinkers if they consumed more than one per day.

We also obtained the numbers of lifestyle- and disease-related risk factors in both age groups. The lifestyle-related risk factors included current smoking, heavy alcohol consumption, and obesity. The disease-related risk factors included hypertension, diabetes mellitus, dyslipidemia, and heart disease.

### 2.4. Statistical Analysis

We compared the age groups using the two-sample t-test for continuous variables and the Chi-square test for categorical variables. *p* < 0.001 indicated statistical significance. Statistical analyses were conducted using SPSS (SPSS Inc., Chicago, IL, USA).

## 3. Results

### 3.1. Baseline Characteristics

Of the total subjects, 915 (8.6%) were aged 19 to 45, inclusive (younger group) (Table 1). The mean age of the younger group was 38.8 ± 5.8 years and that of the older group was 67.6 ± 11.1 years. The proportion of men was significantly higher in the younger group than in the older group (67.7% vs. 55.8%; *p* < 0.001).

**Figure 2:**
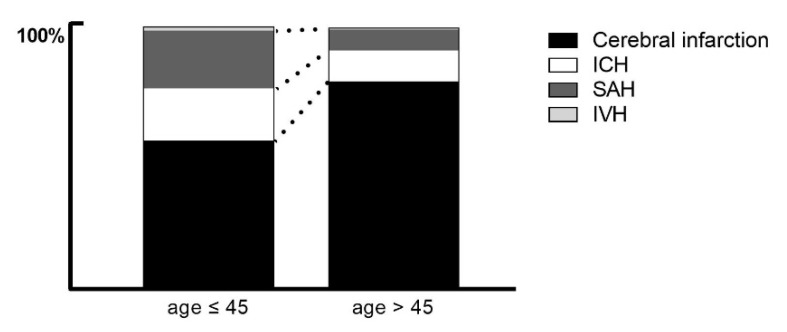
Prevalence of stroke type by age group. Graph shows the prevalence of cerebral infarction (black), ICH (white), SAH (dark gray), and IVH (gray). ICH, intracerebral hemorrhage; SAH, subarachnoid hemorrhage; IVH, intraventricular hemorrhage.

Statistically significant differences in stroke types were found between the younger and older groups. The proportion of hemorrhagic stroke was significantly higher in the younger group than in the older group (44.5% vs. 21.4%; *p* < 0.001). Of the hemorrhagic strokes, ICH and SAH were significantly more common in the younger group than in the older group (*p* < 0.001), but IVH was not (Figure 2). The proportion of vascular abnormality was also significantly higher in the younger group (23.0% vs. 8.0%; *p* < 0.001).

Pre-stroke mRS was significantly lower in the younger group than in the older group (*p* = 0.001), and the Charlson Comorbidity Index—both the weighted index of comorbidity and the combined condition and age-related score—was significantly lower in the younger group than in the older group (both *p* < 0.001). In ischemic stroke, the younger group’s initial NIHSS score was significantly lower than that of the older group (*p* < 0.001), but no significant difference in initial GCS was found for hemorrhagic stroke (Table 1). The time from onset to ER did not differ between the groups.

### 3.2. Risk Factors

**Figure 3:**
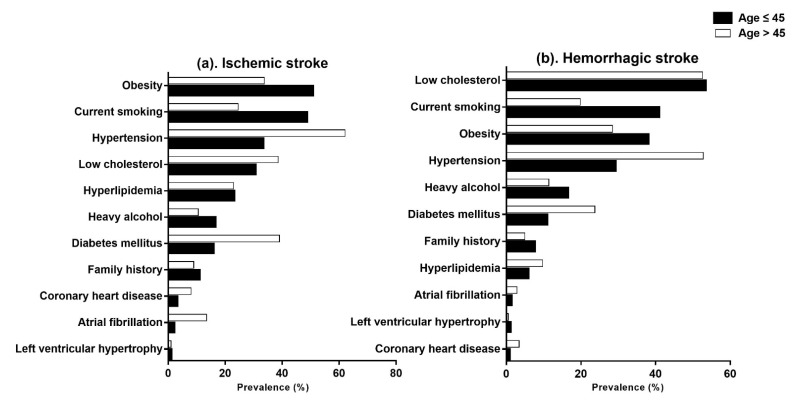
Prevalence of risk factors in the younger group (black) and older group (white), stratified by stroke type: (**a**) ischemic stroke and (**b**) hemorrhagic stroke.

Overall, the frequent risk factors in the younger group were obesity (44.8%), current smoking (44.8%), and low cholesterol (40.1%) in order, but those in the older group were hypertension (60.2%), low cholesterol (41.5%) and diabetes mellitus (35.9%) in order (Table 2). The risk factors that were significantly more frequent in the younger group compared with the older group were obesity (44.8% vs. 32.6%), current smoking (44.8% vs. 23.6%), and heavy alcohol consumption (16.1% vs. 10.7%) (*p* < 0.001) (Figure 3). By contrast, the risk factors that were significantly more frequent in the older group than in the younger group were hypertension (60.2% vs. 31.1%), diabetes mellitus (35.9% vs. 13.3%), dyslipidemia (20.3% vs. 15.2%), atrial fibrillation (11.3% vs. 1.3%), and coronary heart disease (7.1% vs. 1.7%) (*p* < 0.001). We found no significant differences between the younger and older groups in left ventricular hypertrophy (0.7% vs. 0.9%), peripheral artery disease (0.7% vs. 0.9%), low cholesterol (40.1% vs. 41.5%), or family history (9.1% vs. 8.1%) (Table 2).

In ischemic stroke, the frequent risk factors in the younger group were obesity (50.3%), current smoking (48.2%), and hypertension (32.9%) in order, but those in the older group were hypertension (62.1%), diabetes mellitus (39.0%) and low cholesterol (38.6%) in order (Table 2) (Figure 3). Obesity, current smoking, and heavy alcohol consumption were significantly more frequent in the younger group than in the older group (*p* < 0.001). By contrast, hypertension, diabetes mellitus, low cholesterol, atrial fibrillation, and coronary heart disease were significantly more frequent in the older group than in the younger group (*p* < 0.001). We found no significant differences in hyperlipidemia, left ventricular hypertrophy, peripheral artery disease, or family history between the younger and older groups (Table 2).

In hemorrhagic stroke, the frequent risk factors in the younger group were low cholesterol (53.0%), current smoking (40.5%), and obesity (37.7%) in order, but those in the older group were hypertension (52.8%), low cholesterol (52.6%) and obesity (28.5%) in order (Table 2) (Figure 3). Current smoking, obesity, and heavy alcohol drinking were also significantly more frequent in the younger group than in the older group (*p* < 0.001). Hypertension, diabetes mellitus, hyperlipidemia, coronary heart disease, and atrial fibrillation were significantly more frequent in the older group than in the younger group (*p* < 0.001). We did not find any significant differences in low cholesterol, left ventricular hypertrophy, peripheral artery disease, or family history between the younger and older groups (Table 2).

**Figure 4:**
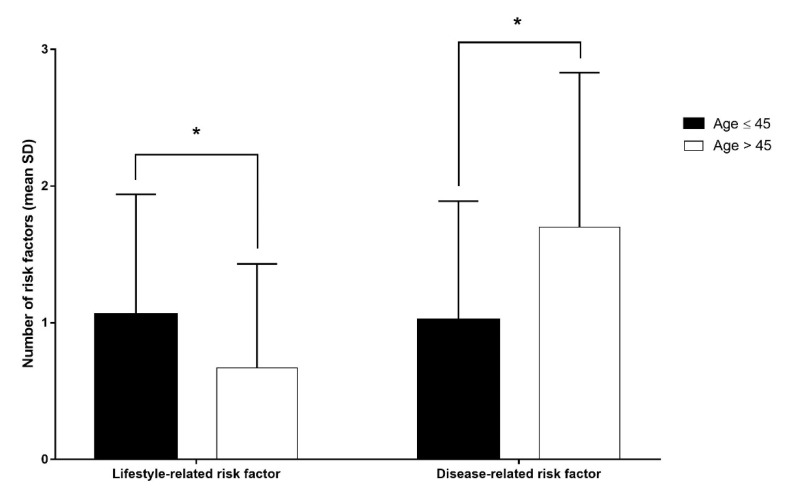
Number of risk factors in the younger group (black) and older group (white). * *p* < 0.001 indicated statistical significance using the two-sample t-test.

The number of lifestyle-related risk factors was significantly higher in the young group than in the older group (*p* < 0.001), whereas the number of disease-related risk factors was significantly lower in the younger group than in the older group (*p* < 0.001) (Figure 4).

## 4. Discussion

The results of this large-scale, multicenter cohort study demonstrate that the clinical characteristics and risk factors of stroke in young adults differ from those in older adults.

In this study, 8.7% of all strokes occurred in patients aged 19–45 years. This is a higher proportion than that reported by Bevan et al., who found that 4.9% of all strokes occurred in patients aged 18–44 in 1990 [4], and Kissela et al., who found that 6.4% of all strokes occurred in patients aged 20–44 in 2005 [9]. However, this result is in line with a more recent study by Maaijwee et al., which showed that roughly 10% of strokes occurred in individuals under the age of 50 in 2014 [5]. This increase in young stroke is also consistent with the results of Kissela et al., whose population-based stroke epidemiology study showed that stroke incidence in younger adults increased over time, most notably between 1999 and 2005 [9]. It may be caused by the recent rise in the prevalence of vascular risk factors in young adults. The US National Health and Nutrition Examination Survey reported an increased prevalence of diabetes, hypercholesterolemia, and obesity in young adults from 1988 to 2006 [1]. A Korean epidemiologic study also showed an increased prevalence of obesity, lack of physical activity, high-risk alcohol consumption, and current smoking in young men aged 19–39 from 1998 to 2012 [24].

We found that men outnumbered women among the young adults who experienced stroke in this study. Although gender disparities in the incidence of stroke are still inconsistent across studies, our findings are consistent with previous studies of stroke in young adult populations [4,14,25,26,27]. The strongly deviated sex ratio may be attributed to different degrees of exposure to risk factors between genders [25]. Previous studies of health behaviors among young men have shown that men are more exposed than women to risk factors such as hypertension, cigarette smoking, alcohol consumption, and high salt intake [25]. Moreover, an increasing trend of obesity was shown in men aged 40 and under but not in women of the same age group from 1998 to 2009 [28].

This study further found that the initial severity of ischemic stroke in the younger group was milder compared with that of the older group, as reported in most previous studies [29,30]. Despite adjusting for baseline differences in stroke risk factors and other comorbidities, Huggins et al. also reported that young adults tend to have lower initial NIHSS [30]. However, the degree to which age-related variations in clinical outcomes point to differences in other factors, such as pathophysiology, comorbid conditions, or divergent responses to treatment, remains unclear [31]. Notably, no significant difference in initial hemorrhagic stroke severity was found between the age groups in this study. This finding is similar to that of Andersen et al., who found that age was not related to stroke severity at admission in hemorrhagic stroke patients [32]. It may be relevant that lesion size in patients with hemorrhagic stroke is generally larger than that of patients with ischemic stroke [32,33].

In this study, hemorrhagic stroke was more frequent in the younger group than in the older group. The proportions of stroke types in the younger group were ischemic stroke (56.5%), SAH (22.2%), and ICH (20.0%) (Figure 2). These findings are similar to those of Marini et al., who found that 57.3% of young adult stroke patients had a cerebral infarction, 22.5% experienced a SAH, and 20.2% experienced an ICH [14]. Our findings are also consistent with most previous studies showing that hemorrhagic stroke occurred in a higher proportion of strokes in young adults (40–55%) compared with the total stroke population (15–20%) [4,14]. This difference in the proportions of stroke types may arise due to a different and wider etiological and risk factor spectrum in younger patients compared with older patients [26,34]. Koivunen et al. also reported that ICH was far more often attributed to structural lesions, such as arteriovenous malformation and cavernous hemangioma, in younger patients than in older adult patients [34]. Similarly, another previous study showed that 48% of normotensive patients who were 45 years of age or younger had angiography abnormalities, whereas hypertensive patients who were older than 45 years had no underlying vascular abnormalities [35].

In this study, obesity (44.8%), current smoking (44.8%), and heavy alcohol drinking (16.1%) were significantly more frequent in the younger group than in the older group, whereas hypertension (60.2%), diabetes mellitus (35.9%), dyslipidemia (20.3%), atrial fibrillation (11.3%), and coronary heart disease (7.1%) were significantly more frequent in the older group. In Korea, there are two major, ongoing, nationwide surveys dealing with the health issues of the greater population: the Korean National Health and Nutrition Examination Survey (KNHANES) has assessed the health and nutritional status of Koreans since 1998 [36], and the National Health Insurance Service-Health Screening Cohort (NHIS-HEALS) is a cohort of Korean participants who participated in health screening programs provided by the NHI [37]. In young adults, the prevalence of obesity and current smoking in this study (both 44.8%) was higher than those reported by KNHANES (28.9%, 22.5%) and NHIS-HEALS (32.2%, 29.3%); the prevalence of heavy alcohol consumption in this study (16.1%) was also higher than that of KNHANES (13.4%) [36,37]. These findings suggest that the most common risk factors of stroke in young adults may be lifestyle-related risk factors, such as smoking, alcohol, and obesity. “These observations are consistent with the recent published data related to the profile of risk factors in young patients with stroke [27,38,39]”.

This study found the frequent risk factors of ischemic stroke in younger adults to be obesity (50.3%), current smoking (48.2%), hypertension (32.9%), and moderate or heavy alcohol consumption (16.1%). Among these risk factors, obesity, smoking, and alcohol consumption were significantly more frequent in the younger group than in the older group, which is in alignment with most previous studies. The regional Australian perspective study reported that smoking (60%), hypertension (36.7%), and dyslipidemia (23.3%) were the most common risk factors of ischemic stroke in patients aged 18–50 years [27]. The Helsinki Young Stroke Registry study reported that dyslipidemia (59.5%), smoking (44.2%), hypertension (39.1%), heavy drinking (14.2%), and obesity (10.6%) were common risk factors in their population of ischemic stroke patients aged 18–49 years [40]. The Young Fabry Patients study also reported that smoking (55.5%), physical inactivity (48.2%), arterial hypertension (46.6%), dyslipidemia (34.9%), and obesity (22.3%) were the most common risk factors in their ischemic stroke and transient ischemic stroke patients aged 18–55 years, inclusive [41].

In hemorrhagic stroke, the most frequent risk factors in the younger group were low cholesterol (53.0%), current smoking (40.5%), obesity (37.7%), hypertension (28.9%), and heavy alcohol consumption (16.1%). Among these factors, current smoking, obesity, and heavy alcohol consumption were significantly more frequent in the younger group than in the older group, which is similar to the findings of most previous studies. Ruiz-Sandoval et al. reported that the most frequent risk factors in ICH patients under 40 years were tobacco use (20%), low cholesterol (35%), hypertension (13%), and alcohol use (10%) [26]. Koivunen et al. reported that the most prevalent risk factors were hypertension (29.8%), smoking (22.3%), and use of alcohol (14.6%) in young stroke patients aged 16–49 years [34]. Li et al. reported that hypertension, alcohol consumption, and smoking were major controllable risk factors for ICH in young adults [42]. Furthermore, Broderick et al. reported that current cigarette smoking, hypertension, and cocaine use were important modifiable risk factors for SAH in young adults [43].

These studies supported our finding that the number of lifestyle-related risk factors was significantly higher and the number of disease-related risk factors was significantly lower in the younger group than in the older group. Therefore, although the identification of risk factors for stroke was complicated by the variation in types of stroke, our results suggest that stroke in young adults was not related to pre-existing disease but rather lifestyle-related risk factors, such as smoking, obesity, and alcohol consumption, regardless of stroke type.

These findings are consistent with most previous studies [39,41,44]. Ferro et al. reported that traditional risk factors for stroke, such as hypertension and diabetes, were not very frequent in young adults; however, other risk factors such as smoking, use of oral contraceptives, migraine, trauma, use of illicit drugs, and pregnancy or puerperium had a more important role in this age group than in older adults [44]. Sarnowski et al. also reported that modifiable risk factors linked to lifestyles, such as smoking, physical inactivity, obesity, and high-risk alcohol consumption, were among the most prevalent in young ischemic stroke [41]. Bailey et al. reported similar results, that the prevalence and adjusted odds ratios for lifestyle risk factors were higher in individuals with stroke than in those without stroke [39]. Our findings are also in line with the Helsinki Young Stroke Registry study, which suggested that the traditional risk factor profile and etiology started to merge those seen in older patients already in early midlife, while this shift accelerates at around age 44 [40].

Lifestyle-related risk factors (smoking, physical activity, alcohol consumption, and obesity) may play an important role in the development of traditional disease-related vascular risk factors in young stroke patients. This finding is supported by Sarnowski et al., who suggested that unfavorable behavioral patterns may provide the first link in a cause-and-effect chain that promotes the development of well-documented vascular risk factors, such as arterial hypertension, diabetes mellitus, or hyperlipidemia. These findings emphasize the need for increased awareness of modifiable risk factors in the principal prevention of stroke in young adult patients. Lifestyle-related risk factors are modifiable, and lifestyle behavioral modifications can reduce stroke. Thus, there is a need for vigorous primary and secondary prevention measures in young populations targeting modifiable lifestyle vascular risk factors [40]. This conclusion is consistent with the American Heart Association recommendations that lifestyle risk factors should be discussed with patients and modified where possible to reduce cardiometabolic risk [45].

This study revealed the typical clinical characteristics and risk factors for stroke in young adults compared with older adults. However, there are some limitations. First, because this study was not a case–control cohort study but an observational cohort study, the frequency of risk factors was obtained instead of odds ratios. Therefore, the data of two major Korean nationwide surveys, which met the definition of risk factors in this study, were also compared to minimize the errors in this study. Second, the collection of broader data on lifestyle-related potential risk factors, such as physical activity, illicit drug use, oral contraceptive use, diet, income, pregnancy, sleep patterns, migraines, etc., was limited because data were obtained retrospectively from the KOSCO prospective cohort study, which has a fixed data structure. Third, we could not analyze vascular abnormalities, which have been suggested as a major factor in young stroke patients, because of insufficient data. A large-scale, prospective, long-term, case–control cohort study of potential risk factors for young adults could provide clearer conclusions. Despite these limitations, the major strength of the study is that the data were obtained from a sufficiently large cohort of adults with stroke to generate reliable estimates of risk factors.

## 5. Conclusions

This study is one of few Asian retrospective studies using large cohort data that present the clinical characteristics and risk factors for stroke in young adults by stroke type. The younger group accounted for 8.6% of all strokes and consisted of more men than women. Ischemic stroke accounted for the largest proportion in the younger group, but the proportions of ICH and SAH were also significantly higher in the younger group than in the older group. The major risk factors for young stroke may be linked to lifestyle and preventable factors. Therefore, increasing awareness of lifestyle-related risk factors may be necessary to prevent stroke in young adults.

## Figures and Tables

**Table 1:** Baseline characteristics of subjects.

	Age ≤ 45 (n = 915)	Age > 45 (n = 9669)	Total (n = 10,584)	*p*-Value
Age (years)	38.8 ± 5.9	67.6 ± 11.1	65.1 ± 13.5	0.000
Sex (Men)	619 (67.7)	5392 (55.8)	6011 (56.8)	0.000
Duration from onset to ER (hours)	0.8 ± 1.5	0.8 ± 1.6	0.8 ± 1.6	0.534
Affected side				0.000
Right	306 (33.4)	3989 (41.3)	4295 (40.6)	
Left	559 (61.1)	5011 (51.8)	5570 (52.6)	
Both	50 (5.5)	669 (6.9)	719 (6.8)	
Location				0.000
Supratentorial	505 (55.2)	6875 (71.1)	7380 (69.7)	
Infratentorial	174 (19.0)	1636 (16.9)	1810 (17.1)	
Both	27 (3.0)	381 (3.9)	408 (3.9)	
Undetermined	209 (22.8)	777 (8.0)	986 (9.3)	
Vascular abnormality ^a^	210 (23.0)	779 (8.1)	980 (9.3)	0.000
Pre-stroke mRS	0.6 ± 1.3	0.7 ± 1.4	0.7 ± 1.4	0.001
Charlson Comorbidity Index				
Weighted index of comorbidity	2.6 ± 1.0	2.8 ± 1.2	2.7 ± 1.2	0.000
Combined condition and age-related score	3.1 ± 1.2	5.3 ± 1.8	5.1 ± 1.9	0.000
Initial NIHSS ^b^	3.4 ± 4.0	5.5 ± 5.9	5.4 ± 5.8	0.000
Initial GCS ^c^	12.0 ± 4.0	12.1 ± 3.9	12.1 ± 3.9	0.886

Categorical data are represented as n (%) and continuous variables are represented as mean ± standard deviation. ^a^ Aneurysm, sarteriovenous malformation, or moyamoya disease. ^b^ Only ischemic stroke evaluated. ^c^ Only hemorrhagic stroke evaluated. ER, emergency room; mRS, modified Rankin Scale; NIHSS, National Institutes of Health Stroke Scale; GCS, Glasgow Coma Scale.

**Table 2:** Prevalence of risk factors of stroke by age group.

	Cerebral Infarction			Cerebral Hemorrhage			Total		
	Age ≤ 45	Age > 45	*p*-Value	Age ≤ 45	Age > 45	*p*-Value	Age ≤ 45	Age > 45	*p*-Value
Hypertension	170 (32.9)	4776 (62.1)	0.000	115 (28.9)	1042 (52.8)	0.000	285 (31.1)	5818 (60.2)	0.000
Diabetes mellitus	80 (15.5)	3003 (39.0)	0.000	42 (10.6)	469 (23.8)	0.000	122 (13.3)	3472 (35.9)	0.000
Heart disease									
Coronary heart disease	14 (2.7)	621 (8.1)	0.000	2 (0.5)	68 (3.4)	0.002	16 (1.7)	689 (7.1)	0.000
Atrial fibrillation	8 (1.5)	1035 (13.5)	0.000	4 (1.0)	56 (2.8)	0.034	12 (1.3)	1091 (11.3)	0.000
Left ventricular hypertrophy	3 (0.6)	75 (1.0)	0.371	3 (0.8)	11 (0.6)	0.641	6 (0.7)	86 (0.9)	0.467
Dyslipidemia									
Hyperlipidemia	117 (22.6)	1768 (23.0)	0.857	22 (5.5)	192 (9.7)	0.008	139 (15.2)	1960 (20.3)	0.000
Low cholesterol	156 (30.2)	2970 (38.6)	0.000	211 (53.0)	1038 (52.6)	0.875	367 (40.1)	4008 (41.5)	0.430
Obesity	260 (50.3)	2593 (33.7)	0.000	150 (37.7)	562 (28.5)	0.000	410 (44.8)	3155 (32.6)	0.000
Smoking			0.000			0.000			0.000
Current	249 (48.2)	1891 (24.6)		161 (40.5)	390 (19.8)		410 (44.8)	2281 (23.6)	
Past	40 (7.7)	1083 (14.1)		13 (3.3)	101 (5.1)		53 (5.8)	1184 (12.2)	
None	228 (44.1)	4721 (61.4)		224 (56.3)	1483 (75.1)		452 (49.4)	6204 (64.2)	
Alcohol consumption			0.000			0.000			0.000
None	212 (41.0)	5015 (65.2)		149 (37.4)	1278 (64.7)		361 (39.5)	6293 (65.1)	
Moderate	222 (42.9)	1868 (24.3)		185 (46.5)	470 (23.8)		407 (44.5)	2338 (24.2)	
Heavy	83 (16.1)	812 (10.6)		64 (16.1)	226 (11.4)		147 (16.1)	1038 (10.7)	
Family history	54 (10.4)	689 (9.0)	0.253	29 (7.3)	98 (5)	0.060	83 (9.1)	787 (8.1)	0.327
Total	517	7695		398	1974		915	9669	

Data are represented as n (%).

## Data Availability

The study data cannot be accessed publicly per the internal regulations of the Korean National Institute of Health because KOSCO (Korean Stroke Cohort for Functioning and Rehabilitation) is an ongoing project.

## Abbreviations

The following abbreviations are used in this manuscript:

ER	Emergency room
GCS	Glasgow Coma Scale
ICH	Intracerebral hemorrhage
IVH	Intraventricular hemorrhage
KNHANES	Korean National Health and Nutrition Examination Survey
KOSCO	Korean Stroke Cohort for Functioning and Rehabilitation
mRS	Modified Rankin Scale
NHIS-HEALS	National Health Insurance Service-Health Screening Cohort
NIHSS	National Institute of Health Stroke Scale
SAH	Subarachnoid hemorrhage
